# Identification of new interaction partners of the human ABC transporter MDR3

**DOI:** 10.1186/2047-783X-19-S1-S19

**Published:** 2014-06-19

**Authors:** Marianne Kluth, Katja Döhl, Philipp Ellinger, Susanne Przybylla, Sander Smits, Lutz Schmitt

**Affiliations:** 1 Institute of Biochemistry, Heinrich Heine University, 40225 Düsseldorf, Germany

## 

The human multidrug resistance protein 3 (MDR3/ABCB4) belongs to the ATP binding cassette (ABC) transporter family found in all kingdoms of life. MDR3 is located in the canalicular membrane of hepatocytes and translocates phosphatidylcholine (PC) from the inner to the outer leaflet of the canalicular membrane energized by ATP hydrolysis. PC is one major component of bile and is essential to protect the biliary duct from bile salts, which are translocated by the bile salt export pump (BSEP/ABCB11). Phosphatidylcholine, bile salts and cholesterol translocated by ABCG5/G8 form mixed micelles. These mixed micelles have a lower capacity to extract lipids from the membrane and prevent the crystallization of cholesterol in the biliary duct. Different types of liver diseases e.g. progressive familial intrahepatic cholestasis type 3 (PFIC 3) are caused by dysfunction of MDR3. On the one hand mutations within the MDR3 gene can abolish the ATPase activity and PC lipid transport, respectively. On the other hand defects of the regulation pathway with respect to the targeting and retention of MDR3 to the canliculare membrane can occur. Currently, it is not known, how MDR3 is regulated and targeted to the canalicular membrane. Further, MDR3 shares over 75 % identity with the multidrug resistance protein 1 (MDR1/ABCB1). MDR1 is well-characterized and plays an enormous challenge in chemotherapy. If we identify the regulation mechanism of MDR3, we will as well contribute to the understanding of MDR1 regulation.

Several adaptor proteins are identified, which are important for the incorporation, retention and degradation of ABC transporters into the canalicular membrane of hepatocytes. For example, the adaptor protein NHERF-1 is crucial for the internalization of MRP2 and MRP4, Radixin (RDX) for MRP2 and HAX-1 for BSEP. NHERF-1 contains two PDZ domains, which binds amongst others the highly conserved motive (S/T)X(V/I/L/A/F/M) (X = any amino acid). The primary sequence of the second MDR3 nucleotide binding domain (NBD) contains several times this binding motive. This is a first hint that NHERF-1 binds also MDR3. RDX is a member of the Ezrin/Radixin/Moesin (ERM) protein family and is mainly localized at the canalicular membrane of hepatocytes. The N-terminale FERM (4.1-protein, Erzin, Radixin, Moesin) domain of RDX binds the C-terminus of MRP2. The amino acid sequence alignment of MRP2 and MDR3 showed no conserved amino acids at the C-terminus.

Ikebuchi Y. *et al.* used a yeast-two hybrid screen with a cytoplasmatic linker region of MDR3 and a human liver cDNA library to identify interaction proteins of MDR3. They determined that the localization of MDR3 is regulated by the receptor for activated C-kinase 1 (RACK1) [1]. We used RACK1 as an internal control for our experiments.

To understand how MDR3 is targeted to the apical membrane, we aimed to identify further interaction partners of this transporter. For this purpose we want to use *in vitro* pulldown assays, size exclusion chromatography and blue native page with purified MDR3 and the potentially interaction proteins. First, we cloned the human cDNA of MDR3 in *Saccharomyces cerevisiae* and expressed *MDR3* in the methylotrophic yeast *Pichia pastoris* in amounts suitable for a detailed structure-function analysis. We tested over 100 different detergents via dotblot analysis and found that only lipid-like detergents as Fos-cholines (FCs) were able to extract MDR3 in adequate yields. MDR3 was purified with FC-16 via tandem-affinity purification (TAP) consisting of an immobilized metal ion affinity chromatography and a calmodulin binding peptide purification step. About 6 mg protein per 100 g wet cells were obtained with high purity and homogeneity. The functionality was determined by measuring the ATPase activity of purified MDR3 in detergent solution. Purified MDR3 exhibited significant ATP hydrolysis activity that could be stimulated by DPPC and DOPC. To eliminate any possibility of unspecific ATPase activity due to contaminants, we cloned and purified an ATPase inactive mutant, where the glutamic acid is changed to a glutamine in the highly conserved Walker B motive of both NBDs. This mutant (E558Q/E1027Q) showed basal ATPase activity comparable to the wild-type protein. But more important, no ATPase activity stimulation by adding PC lipids to the reaction was obtained. So far our work demonstrates that heterologously expressed MDR3 can be purified in a functional state with respect to its capability to bind and hydrolyze ATP [2].

Second, we cloned the cDNA of the adaptor proteins NHERF-1 and RDX combined with a StrepII affinity tag in the *Escherichia coli* expression system. RACK1 cDNA was fused to a hexa-histidine affinity tag and expressed also in *E. coli*. Recently, we purified the interaction proteins NHERF-1 and RDX with Strep-tactin sepharose to high purity and homogenity. RACK1 was purified via immobilized metal ion affinity purification and size exclusion chromatography. We obtained approximately 20 mg out of 2 L cell culture.

To determine the interaction of MDR3 with the putative interaction partners NHERF-1, RDX and RACK1 we want to use the *in vitro* pull-down assay, size exclusion chromatography (SEC) and blue native page. For the pull-down assay, the purified interaction partner was mixed with the purified and functional MDR3. Then one of the proteins was immobilized on the corresponding matrix by the affinity tag. After several washing steps, the complex was eluted and identified via immunoblotting. Currently, we have done initial pull-down assays with MDR3 and the positive control RACK1. MDR3 was immobilized by the calmodulin binding peptide (CBP) to calmodulin resin and eluted after adding EGTA. RACK1 has a size of 35 kDA and is obtained at the 35 kDA marker band in the SDS-PAGE gel. It binds unspecifically to the CBP resin. In the immunoblot against RACK1 we determined in the reaction of MDR3 and RACK1 an additional band of about 130 kDA for RACK1, while RACK1 alone ran at 35 kDa. One explanation is that RACK1 forms a highly stable complex with MDR3, which is resistent against SDS. To confirm this result we tried blue native page, but unfortunately we did not succeed so far. With this first results we can verify that MDR3 interacts with RACK1. Further, we want to analyze NHERF-1 and RDX concerning the potential interaction with MDR3.
